# Methanogenesis in biogas reactors under inhibitory ammonia concentration requires community-wide tolerance

**DOI:** 10.1007/s00253-023-12752-5

**Published:** 2023-09-06

**Authors:** Damien R Finn, Lena Rohe, Sascha Krause, Jabrayil Guliyev, Achim Loewen, Christoph C. Tebbe

**Affiliations:** 1 Thünen Institute for Biodiversity, Johann Heinrich von Thünen Institute, 38116 Braunschweig, Germany; 2 Thünen Institute for Climate-Smart Agriculture, Johann Heinrich von Thünen Institute, 38116 Braunschweig, Germany; 3 School of Ecological and Environmental Sciences, East China Normal University, Shanghai, 200062 China; 4 Faculty of Resource Management, University of Applied Sciences and Arts (HAWK), 37085 Göttingen, Germany

**Keywords:** Biogas, Anaerobic digestion, Ammonia inhibition, Molecular community ecology, Methanogenic *Archaea*

## Abstract

**Abstract:**

Ammonia (NH_3_) inhibition represents a major limitation to methane production during anaerobic digestion of organic material in biogas reactors. This process relies on co-operative metabolic interactions between diverse taxa at the community-scale. Despite this, most investigations have focused singularly on how methanogenic *Archaea* respond to NH_3_ stress. With a high-NH_3_ pre-adapted and un-adapted community, this study investigated responses to NH_3_ inhibition both at the community-scale and down to individual taxa. The pre-adapted community performed methanogenesis under inhibitory NH_3_ concentrations better than the un-adapted. While many functionally important phyla were shared between the two communities, only taxa from the pre-adapted community were robust to NH_3_. Functionally important phyla were mostly comprised of sensitive taxa (≥ 50%), yet all groups, including methanogens, also possessed tolerant individuals (10–50%) suggesting that potential mechanisms for tolerance are non-specific and widespread. Hidden Markov Model–based phylogenetic analysis of methanogens confirmed that NH_3_ tolerance was not restricted to specific taxonomic groups, even at the genus level. By reconstructing covarying growth patterns via network analyses, methanogenesis by the pre-adapted community was best explained by continued metabolic interactions (edges) between tolerant methanogens and other tolerant taxa (nodes). However, under non-inhibitory conditions, sensitive taxa re-emerged to dominate the pre-adapted community, suggesting that mechanisms of NH_3_ tolerance can be disadvantageous to fitness without selection pressure. This study demonstrates that methanogenesis under NH_3_ inhibition depends on broad-scale tolerance throughout the prokaryotic community. Mechanisms for tolerance seem widespread and non-specific, which has practical significance for the development of robust methanogenic biogas communities.

**Key points:**

• *Ammonia pre-adaptation allows for better methanogenesis under inhibitory conditions.*

• *All functionally important prokaryote phyla have some ammonia tolerant individuals.*

• *Methanogenesis was likely dependent on interactions between tolerant individuals.*

**Supplementary Information:**

The online version contains supplementary material available at 10.1007/s00253-023-12752-5.

## Introduction

Biogas is becoming a widespread means for producing heat, electricity and fuel energy from non-fossil fuel sources. It is the end result of prokaryotic anaerobic digestion (AD) of organic matter, with methane (CH_4_) acting as the primary energy-rich component. Currently, most biogas is generated from agricultural waste, manure and energy crops, e.g. maize, straw (Scarlat et al. 2018). Co-digestion of waste and/or energy crops with manure is favourable for maximising CH_4_ yields (Scarlat et al. 2018); however, protein hydrolysis of nitrogen-rich manure results in the accumulation of ammonia (NH_3_) and potential inhibition of the AD process (Chen et al. 2008; Venkiteshwaran et al. 2015). An exact mechanism for the inhibitory effect is unknown, although it is speculated that membrane-permeable free NH_3_ enters prokaryotic cells and disrupts intracellular pH, intracellular cation exchange and enzyme function (McCarty and McKinney 1961; Whittmann et al. 1995). Total ammonium nitrogen concentrations (TAN) of greater than 1.7 g L^−1^ are frequently reported as a threshold for significant inhibition of AD (Yenigün and Demirel 2013). However, as it is specifically the un-ionised NH_3_ that is toxic, AD inhibition can occur at concentrations as low as 0.15 g NH_3_ L^−1^ (McCarty and McKinney 1961). Consequently, a myriad of management and technological approaches have been considered to improve manure co-digestion during AD (Fuchs et al. 2018; Yenigün and Demirel 2013).

The AD process occurs in four distinct stages. The first stage of AD involves the catabolism of polymeric organic matter (e.g. plant material) to monomers by specific microbial taxa that produce extracellular hydrolytic enzymes, such as cellulases, lipases and proteases (Wirth et al. 2012). This stage is commonly referred to as hydrolysis (Venkiteshwaran et al. 2015). Next, primary fermenters reduce monomers (e.g. monosaccharides) to carboxylic acids (Devries et al. 1970; Iannotti et al. 1973; Lovley and Phillips 1989), in a stage referred to as acidogenesis. The taxa that perform this step are particularly diverse, and individual taxa can either produce acetate as a waste metabolite or volatile fatty acids (VFAs) with more than two C atoms (> C_2_) (e.g. butyric, propionic, succinic acids). Where these larger VFAs are produced, secondary fermenters further metabolise this to C_1_–C_2_ waste products (de Bok et al. 2001; Hobson et al. 1974; Liu et al. 1999; Thauer et al. 1968) in a stage described as acetogenesis. Finally, diverse methanogenic *Archaea* reduce these C_1_–C_2_ waste products to CH_4_ (Garcia et al. 2000; Thauer et al. 2008) in a stage termed methanogenesis. Importantly, no single taxon is capable of carrying out the four steps of AD (Wirth et al. 2012), and thus anaerobic prokaryotes must work co-operatively to produce the desired end product of CH_4_.

While all prokaryotes in a biogas reactor are likely affected by NH_3_ toxicity, previous studies have primarily focused on methanogens (Borja et al. 1996; Fotidis et al. 2013; Lv et al. 2019; Moestedt et al. 2016; Ziganshin et al. 2016). A general trend appears to be a shift in the dominant methanogenic pathway from aceticlastic to hydrogenotrophic taxa under inhibitory levels of NH_3_ (Gao et al. 2015; Li et al. 2017). The reason for this remains unclear, particularly as only specific hydrogenotrophic taxa are consistently enriched, e.g. *Methanoculleus*, while others are not, e.g. *Methanobacterium* and *Methanobrevibacter* (Moestedt et al. 2016; Ziganshin et al. 2016). Furthermore, facultative and obligate aceticlasts can still be enriched under NH_3_ stress in certain reactors, e.g. *Methanosarcina* and *Methanothrix* (Fotidis et al. 2013; Lv et al. 2019). These seemingly contradictory results have prompted studies to consider broader-scale prokaryote community analyses that note decreases in other functional groups, such as secondary fermenters (Lv et al. 2019). However, a comprehensive understanding of how NH_3_ toxicity affects the prokaryote community as a whole is still lacking.

As the AD process is inherently community-dependent, this study sought to observe the individual growth responses of all taxa to NH_3_ toxicity and, ultimately, how this affected the formation (or loss) of ecological interactions between them. These ecological interactions were used as a proxy to understand the metabolic interactions essential for the continuation of the AD process. This was achieved with a combinatorial experimental design involving (a) two biogas slurry prokaryote communities sourced from either relatively low (un-adapted) or high (pre-adapted) ammonium (NH_4_-N) reactors; and (b) two anaerobic growth media with non-inhibitory or inhibitory levels of NH_3_. Growth responses of the dominant *ca*. 50 phyla were followed over time, in addition to deeper analyses of all individual methanogenic taxa. Finally, the sensitivity and tolerance of each taxon to NH_3_ was determined, and the ecological relationships between sensitive and tolerant taxa were visualised. It was hypothesised that (1) the pre-adapted reactor community would perform methanogenesis better under NH_3_ stress; (2) taxa from all important functional groups, beyond only the methanogens, would be affected by NH_3_ toxicity; and (3) methanogenesis under NH_3_ stress was dependent on the continuation of stable co-operative interactions between taxa.

## Materials and methods

### Sample collection

Prokaryote communities were sourced from two mesophilic (37°C) biogas reactors operating on independent farms in Lower Saxony, Germany. Slurry samples were sealed in wide-mouth LDPE plastic bottles (Kautex, Bonn, Germany) to limit gas diffusion, transported immediately to the lab and stored at −20°C. Chicken manure was sourced from a household with domesticated chickens in Lower Saxony, Germany. Total carbon (%) and nitrogen (%) of slurry and manure samples were determined via dry combustion (LECO TruMac, St. Joseph, MI, USA) on oven-dried (50°C) samples ground to a particle diameter of 50 μm. Total ammonium concentration (g NH_4_-N L^−1^) of samples were determined by continuous-flow analysis (San++ Skalar, Breda, The Netherlands) on 0.45 μm diameter filtered slurry diluted 10^−1^ in distilled water. The pH of both slurries was measured with a HI 2211 pH metre (HANNA Instruments, Vöhringen, Germany). The specific concentration of NH_3_ was calculated as described previously (Fotidis et al. 2013) using a K_a_ dissociation constant of 1.20 × 10^−9^ at 37°C, as:

1 $$\left[{NH}_3\right]=\frac{\left[{NH}_4-N\right]}{1+\frac{10^{- pH}}{K_a}}$$

The NH_4_^+^ and NH_3_ concentrations were assumed to sum to the TAN measured as per continuous-flow analysis. These properties are listed in Table 1. One slurry was considered un-adapted to NH_3_-N (*ca*. 1.2 g TAN L^−1^) and the other pre-adapted (*ca*. 2.3 g TAN L^−1^).

**Table 1:** Chemical and microbial community properties of chicken manure substrate and slurry sources used in this study. Alpha-diversity values based on three replicates. NH_4_^+^-N and NH_3_-N (g per L) were calculated assuming mesophilic biogas temperature conditions of 37°C

Source	C (%)	N (%)	C:N ratio	TAN (g per L)	NH_4_^+^-N (g per L)	NH_3_-N (g per L)	pH	ASV Richness	Pielou Evenness
Chicken manure	31.4	2	15.7	-	-	-	-	230 ± 15	0.7 ± 0
Un-adapted slurry	41.8	3.1	13.5	1.19	1.11	0.07	7.7	544 ± 49	0.84 ± 0
Pre-adapted slurry	34.8	3	11.6	2.3	2.18	0.11	7.6	155 ± 41	0.67 ± 0

### Incubations

Growth incubations were performed in basal anaerobic medium (BAM) prepared as described previously (Angelidaki et al. 1990) in rubber-sealed 125-mL glass serum bottles (Wheaton Industries, Millville, NJ, USA). The pH of media was 6.4 prior to the addition of slurry and manure. Inoculum from either un- or pre-adapted slurries was added to media at a final concentration of 1.5% (w/v) and manure was added at a final concentration of 0.75% (w/v) consistent with VDI-4630 recommendations for batch culture incubations (VDI-4630 2016). The pH after addition of slurry and manure was *ca*. 7.6. After addition of slurry and manure, the BAM had a final NH_3_ concentration of *ca*. 0.09 mg mL^−1^ at 37°C. Excess ammonium chloride (Merck, Darmstadt, Germany) was added to a second BAM to achieve a final NH_3_ concentration of *ca*. 0.3 mg mL^−1^ at 37°C. These were considered relatively low NH_4_-N and relatively high NH_4_-N media, respectively. Incubations were made anaerobic by flushing with pure nitrogen gas at a rate of 160 mL min^−1^ for 15 min, then set under shaking conditions (100 rpm) at 37°C (CH-4103 Infors AG, Bottmingen, Switzerland). The final experimental design consisted of the following slurry *×* media combinations: (1) un-adapted slurry in low NH_4_-N medium; (2) un-adapted slurry in high NH_4_-N medium; (3) pre-adapted slurry in low NH_4_-N medium; and (4) pre-adapted slurry in high NH_4_-N medium. Incubations were destructively sampled after 1, 3, 7 and 14 days. Each treatment and timepoint had three independent replicates.

### Chemical analyses

At each time point, 600 μL of gas headspace was sampled with a gas-tight syringe (Trajan Scientific, Ringwood, Australia) and diluted in pure nitrogen gas within sealed 12-mL glass Exetainer vials (Labco, Buckinghamshire, UK). Gas pressure before and after sample addition was measured (GDH 12 AN, Greisinger, Regenstauf, Germany) in order to calculate a dilution factor. Carbon dioxide (CO_2_) and CH_4_ concentrations were determined with an 7890A GC System (Agilent, Waldbronn, Germany), with a helium ionisation detector, and helium as a carrier gas at a constant flow rate of *ca*. 12 mL min^−1^. Standards for CO_2_ included 0, 350, 704, 1500, 1999 and 3012 ppm, and for CH_4_ 0, 2.42, 3.43, 4.99, 49.2 and 100.3 ppm (Linde GmbH, Leuna, Germany). Accuracy of GC standard measurements was 98.6 ± 0.02%. Both gases were expressed in units of mL per L of total gases within the headspace volume of the glass serum bottle. At the sampling point, the pH of the incubation was measured with a HI 2211 pH metre (HANNA Instruments, Vöhringen, Germany). Finally, 2 mL of culture was filtered at 0.45 μm diameter, diluted 10^−1^ in distilled water and the NH_4_-N concentration determined via continuous-flow analysis as above (please see the “Sample collection” section). The specific concentration of NH_3_ was calculated as above.

### DNA extraction and molecular analyses

After gas sampling, 1.5 mL of culture was centrifuged at 13,000 rpm for 10 min (Allegra X-15R, Beckman Coulter, Krefeld, Germany) and DNA was extracted from pelleted material with a FastDNA spin kit for soil (MP Biomedicals, Eschwege, Germany). Purified DNA was measured with a NanoDrop 2000c spectrophotometer (Thermo Fisher Scientific, Braunschweig, Germany). From 10 ng μL^−1^ DNA, *Bacteria*, *Archaea* and total Prokaryotes were quantified as described previously (Yu et al. 2005). Briefly, 338F-805R, 787F-1059R and 515f-Parada-806r-Apprill primers were used to target the 16S rRNA gene of the respective groups using SYBR Green Mix in 25 μL reactions with reagent concentrations as per the manufacturer’s instructions (Thermo Fisher Scientific, Waltham, MA, USA) on a Bio-Rad CFX96 real-time PCR system (Bio-Rad Laboratories, Hercules, CA, USA). Thermocycler conditions consisted of 95°C denaturation for 5 min, followed by 40 cycles of 95°C denaturation for 15 s and primer annealing/polymerase extension at 60°C (338F-805R/787F-1059R) or 52°C (515f-Parada-806r-Apprill) for 1 min. Calculations were determined relative to slopes of standard curves (*ca*. 100% efficiency, *R*^2^ > 0.99) over 40 cycles. Community sizes were expressed as 16S rRNA gene copies mL^−1^.

Furthermore, PCR amplification of the 16S rRNA gene V4 hypervariable region was conducted using the 515f-Parada-806r-Apprill primers. This was performed with Q5 DNA polymerase (New England Biolabs, Ipswich, MA, USA) on a Mastercycler X50s (Eppendorf, Wesseling, Germany). Thermocycler conditions consisted of 95°C denaturation for 3 min, followed by 35 cycles of 95°C denaturation for 30 s, primer annealing at 52°C for 30 s, polymerase extension at 72°C for 30 s and a final extension at 72°C for 3 min. Products were confirmed visually by gel electrophoresis PowerPro (Roth, Karlsruhe, Germany) with 1% agarose in 1 *×* Tris-acetate EDTA buffer and stained with Midori Green (Nippon Genetics, Düren, Germany). Amplicon products were subsequently purified with the QIAquick purification kit (Qiagen, Dusseldorf, Germany) and each sample normalised to 1–2 ng μL^−1^ with a SequalPrep Normalisation kit (Thermo Fisher Scientific, Waltham, MA, USA).

Normalised products were sent to LGC Genomics (Berlin, Germany) for the following steps. Briefly, amplicon pools were further purified with one volume Agencourt AMPure XP beads (Beckman Coulter, Brea, CA, USA). Amplicon libraries were prepared with the Ovation Rapid DR Multiplex System (NuGEN Technologies, Redwood City, CA, USA). Illumina adaptors were incorporated into amplicons using PCR (total volume 30 μL) with 12 cycles using MyTaq (Bioline, Luckenwalde, Germany). Fragments of 400–800 bp were gel extracted and purified with MinElute columns (Qiagen, Dusseldorf, Germany) and quality checked via Fragment Analyser (Agilent Technologies, Santa Clara, CA, USA). Sequencing was then performed on the Illumina MiSeq platform (2 *×* 300 bp) (Illumina, San Diego, CA, USA).

### Bioinformatics

Upon receiving amplicon reads, an in-house Python script was used to reorient any forward and reverse reads that had been sequenced in the 3’ orientation (*ca.* 50%) as a consequence of in-house barcoding and normalisation of amplicons (http://github.com/DamienFinn/MiSeq_read_reorientation). Reads were quality filtered, dereplicated and denoised with DADA2 using the Qiime2 pipeline (Bolyen et al. 2019; Callahan et al. 2016) yielding an average of 53,000 observations of amplicon sequencing variants (ASVs) per sample. The ASVs were assigned taxonomy based on the Genome Taxonomy Database release 207 via Qiime2 with the sklearn function under default parameters (Parks et al. 2022). Sequences identified as derived from chloroplasts or mitochondria were filtered from the dataset. Singletons and doubletons were not filtered from the dataset.

Finally, in order to maximise sensitivity and specificity of taxonomic assignment of methanogenic Archaea in the dataset, a novel Hidden Markov Model (HMM) was developed (Eddy 2004). Briefly, the full length 16S rRNA gene of 94 methanogenic taxa from 27 distinct genera (including newly discovered *Candidatus* Methanosuratus, Methanomethylicus and Bathyarchaeota groups) were aligned with MUSCLE (Edgar 2004) and a HMM built as described (Eddy 2011). This HMM was then parsed over all ASVs in this dataset, and identified 92 unique putative methanogenic taxa.

### Statistical analyses

All statistical analyses were conducted in R v 4.0.3 (R Core Team 2013). Change in NH_3_, CO_2_ and CH_4_ production and total *Bacteria* and *Archaea* populations over time were visualised with ggplot2 (Wickham 2016) with trend lines fitted via local regression (loess). Change over time and high vs. low NH_4_-N medium effects were tested with two-way analysis of variance (ANOVA) with a significance level of 0.05.

ASV abundances were initially rarefied with the ‘vegan’ package (Oksanen et al. 2013) to 15,000 observations per sample based on plateauing of rarefaction curves. After conversion of ASVs to relative abundance (%), percentages were weighted with the 16S rRNA gene copies mL^−1^ calculated based on 515f-Parada/806r-Apprill primers in order to derive absolute abundances of each ASV per sample. This was done to avoid mathematical issues related to data proportionality (Gloor et al. 2017). Growth responses of the *ca*. top 50 phyla were visualised as a heatmap (Warnes et al. 2019), with changes over time and high *vs.* low NH_4_-N medium effects on each phylum tested via general linear models. Probability values were Benjamini-Hochberg adjusted to account for false discovery rate (Benjamini and Hochberg 1995) due to the large number of repetitive tests.

Alpha-diversity indices for ASV Richness and Pielou Evenness were calculated for each sample (Oksanen et al. 2013). Initial values for both slurries and manure are provided in Table 1. Change in alpha-diversity over time and effects of high vs. low NH_4_-N medium were tested with two-way analysis of variance. Beta-diversity of communities was visualised via nonmetric multidimensional scaling (NMDS) on Bray-Curtis transformed absolute abundances of individual ASVs per sample. Multivariate Analysis of Similarity (ANOSIM) was used to test for a combined effect of change over time in response to high vs. low NH_4_-N medium (Oksanen et al. 2013).

The sensitivity and tolerance to NH_3_ was calculated for all taxa within this study. Briefly, this was calculated as Hurlbert’s niche breadth in relation to NH_3_ (B_N_-Ammonia) as described previously (Finn et al. 2020). Taxa negatively affected by NH_3_ have a B_N_ approaching 0, while those that are enriched under high concentrations have a B_N_ approaching 1. Significant lower and upper thresholds were as follows: taxa with a B_N_-Ammonia less than 0.24 were significantly negatively impacted by NH_3_ (i.e. sensitive), while taxa above this threshold were tolerant. Taxa with a B_N_-Ammonia greater than 0.88 were significantly positively associated with NH_3_ (i.e. enriched with higher concentrations). These threshold values were calculated based on deriving *p* values from each taxon’s B_N_ relative to a null model distribution consisting of 999 randomly generated taxa (Finn et al. 2020). As above, *p* values were Benjamini-Hochberg adjusted to account for false discovery rate (Benjamini and Hochberg 1995).

For accurate identification of methanogens, the ASVs identified via the HMM as being methanogens underwent phylogenetic analysis. Briefly, the 92 unique putative methanogen ASVs and 94 reference 16S rRNA genes were aligned with MUSCLE in R (Edgar 2004), sequence similarity calculated based on identity and visualised based on neighbour-joining (Schliep et al. 2017).

Finally, covarying growth patterns between taxa over time were calculated via network analyses. Four networks were constructed, one for each of the slurry-medium experimental combinations. Each unique sample was included in network construction in order to maximise reliability of covariations between taxa across samples. The number of input taxa per network was normalised to the 1000 most abundant. Taxa were ASVs. Significant positive Spearman correlations between individuals were determined (*p* < 0.05) (Langfelder and Horvath 2008; Langfelder and Horvath 2012), Benjamini-Hochberg adjusted and visualised with the multidimensional scaling layout in the ‘igraph’ package (Csárdi and Nepusz 2006). To aid comprehension, taxa (as network nodes) were coloured based on their sensitivity (white) or tolerance (grey) to NH_3_, and their strong covarying growth with sensitive methanogens (light blue) or tolerant methanogens (dark blue) were highlighted in the network. Chi-square tests were used to compare whether the abundances of sensitive taxa, sensitive methanogens, tolerant taxa and tolerant methanogens differed between the four networks.

## Results

### Methane production was dependent on pre-adaptation to NH_4_-N

The prepared low NH_4_-N medium had a specific NH_3_ concentration below the lower estimated toxic threshold while the high NH_4_-N medium exceeded it, at 0.09 and 0.3 mg NH_3_ mL^−1^, respectively (Supplementary Figure 1). Over the course of the 14-day incubation period, the specific NH_3_ concentrations in both media decreased (ANOVA, *p* < 0.001) as the pH decreased due to fermentation; however, the high NH_4_-N medium remained above 0.2 mg NH_3_ mL^−1^. Consequently, the following analyses focused upon differential responses of the un-adapted vs. the pre-adapted community to either high or low NH_4_-N media.

**Fig. 1:**
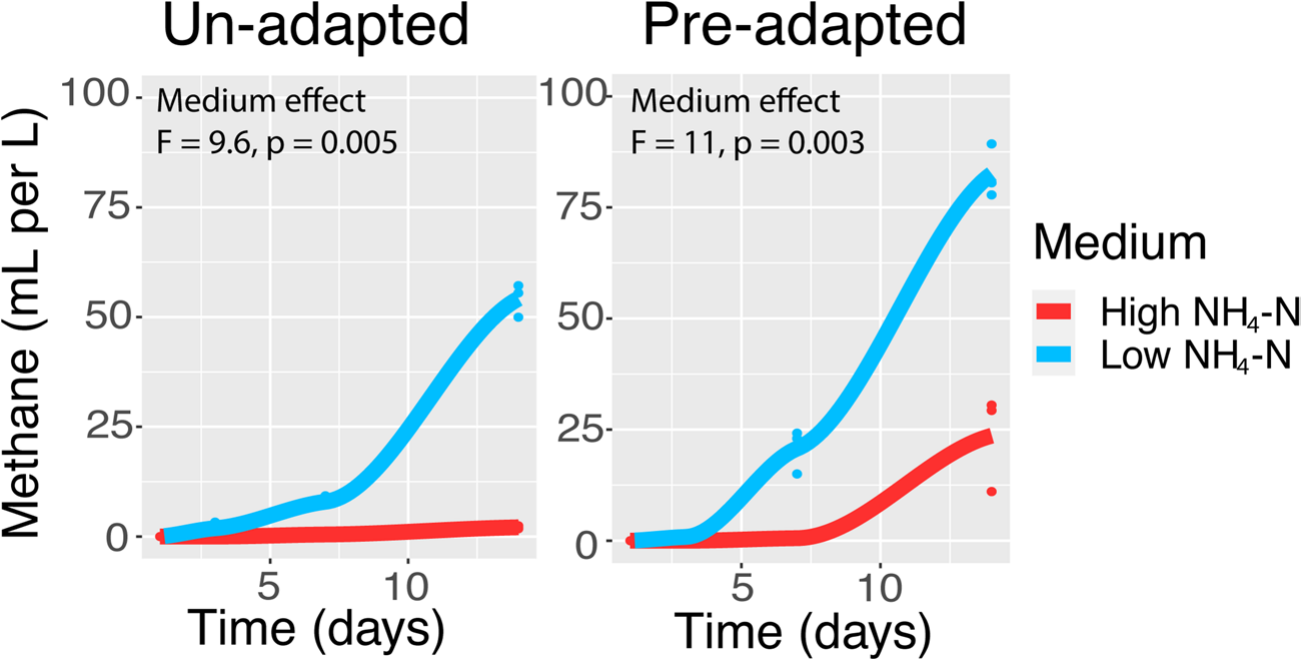
Methane in the gas headspace, produced by un- and pre-adapted communities, over time. Trend lines represent best fit of local regression (loess). Analysis of variance results comparing an effect of low *vs.* high NH_4_-N medium for each community are shown

The un-adapted prokaryote community under the low NH_4_-N medium was able to produce more CH_4_ than under high conditions (*ca*. 54 *versus* 2 mL CH_4_ L^−1^, respectively) (ANOVA, *p* = 0.005) (Fig. 1). While the pre-adapted high NH_4_-N community produced more CH_4_ under high NH_4_-N media than its un-adapted counterpart (*ca*. 25 mL CH L^−1^), methanogenesis was still more efficient in the absence of NH_3_ inhibition (ANOVA, *p* = 0.003). This was reflected in the archaeal population size, with those in the un-adapted community dying over time under high NH_4_-N media (ANOVA, *p* = 0.01) (Supplementary Figure 2). Pre-adapted high NH_4_-N slurry archaeal populations remained stable over time. Neither carbon dioxide respiration nor bacterial population sizes were negatively impacted by high NH_4_-N media, regardless of slurry source (Supplementary Figures 3 and 4).

### Growth of specific phyla was dependent on pre-adaptation to NH_4_-N

**Fig. 2:**
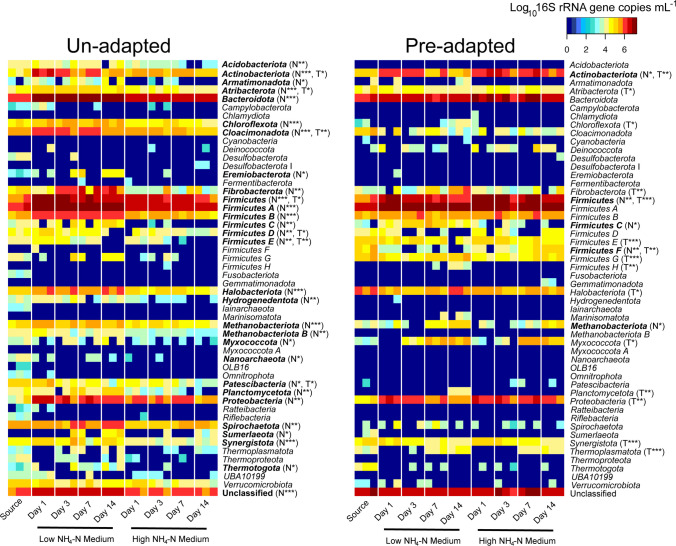
Heatmap of phyla abundances of un- and pre-adapted communities over time. General linear models were used to test an effect of time (T) and NH_4_-N (N) for each community. Benjamini-Hochberg adjusted *p* values are shown as follows: *p = 0.05, **p = 0.001, ***p < 0.001. Phyla affected by NH_4_-N are highlighted in bold

Twenty-nine phyla from the un-adapted slurry were negatively impacted by the high NH_4_-N medium (Fig. 2, bold), representing over half of the identified phyla. These included bacterial *Acidobacteriota*, *Actinobacteriota*, *Bacteroidota*, *Chloroflexota*, *Fibrobacterota*, various phyla split by recent taxonomy from *Firmicutes*, such as *Firmicutes* (Bacilli), *Firmicutes* A (Clostridia), B (Syntrophomonadia), C (Negativicutes) and E (Symbiobacteriia), *Spirochaetota*, *Synergistota* and *Thermotogota*. It also included archaeal *Halobacteriota*, *Methanobacteriota* and *Methanobacteriota* B. In direct contrast, several phyla from the pre-adapted slurry actually grew better in the high NH_4_-N medium (Fig. 2, bold). These included *Actinobacteriota*, *Firmicutes* and *Firmicutes* F (Halanaerobiia). Consistent with the un-adapted phyla, *Firmicutes* C and *Methanobacteriota* from the pre-adapted slurry preferred low NH_4_-N conditions.

**Fig. 3:**
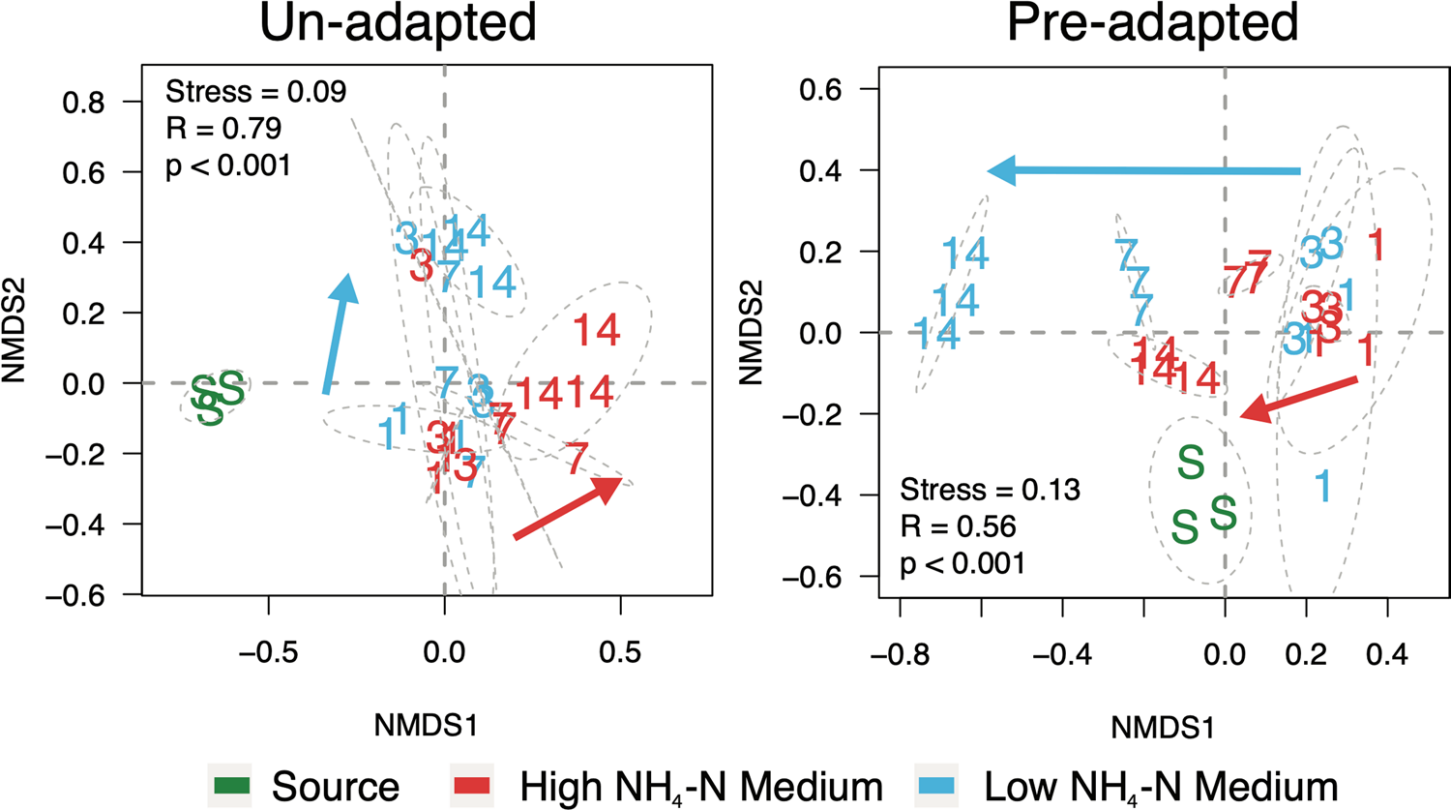
Nonmetric multidimensional scaling of changes in un- and pre-adapted community composition over time. Beta-diversity is based on Bray-Curtis transformation of absolute abundances of ASVs. Sample codes are as follows: S = slurry source; numbers = incubation day of sampling. Colours are based on growth medium or slurry source. Grey dotted lines represent 95% confidence intervals. Analysis of Similarity results testing for changes in community composition over time, between the growth media, are shown. Arrows represent general directionality of compositional shifts

Prokaryote community alpha-diversity followed these general patterns. Firstly, both amplicon sequence variant (ASV) Richness and Pielou Evenness of the un-adapted slurry source were greater than from the high NH_4_-N reactor (Table 1), suggesting greater biodiversity under non-inhibiting NH_3_ conditions. Over the course of the in vitro incubation, the relatively high ASV Richness of the un-adapted community decreased regardless of growth medium (ANOVA, *p* = 0.01) (Supplementary Figure 5). The pre-adapted community Richness remained stable under the incubation conditions, regardless of growth medium. Taxon Evenness was growth medium dependent, whereby the alpha-diversity was stable when the un-adapted community was grown in the low NH_4_-N medium, and the pre-adapted community was grown in the high NH_4_-N medium. In contrast, the un-adapted community decreased in the high NH_4_-N medium (ANOVA, *p* = 0.002) and the pre-adapted community decreased over time in the low NH_4_-N medium (ANOVA, *p* = 0.01) (Supplementary Figure 5). Beta-diversity analyses firstly confirmed that un- and pre-adapted communities were distinct (Supplementary Figure 6). A simultaneous comparison of all communities showed that differences were primarily driven by un- *vs.* pre-adaptation, and secondly by low *vs.* high NH_4_-N medium (ANOSIM, R = 0.86, *p* < 0.001) (Supplementary Figure 7). Finally, a comparison of how each community responded to the media showed that, while communities at days 1 and 3 were similar irrespective of medium, the community composition from the un-adapted slurry diverged distinctly over time depending on the medium (ANOSIM, R = 0.79, *p* < 0.001) (Fig. 3). The composition of the pre-adapted community was more similar to its source under the high NH_4_-N medium (ANOSIM, R = 0.56, *p* < 0.001) (Fig. 3).

### Ammonia sensitivity and tolerance of taxa

**Fig. 4:**
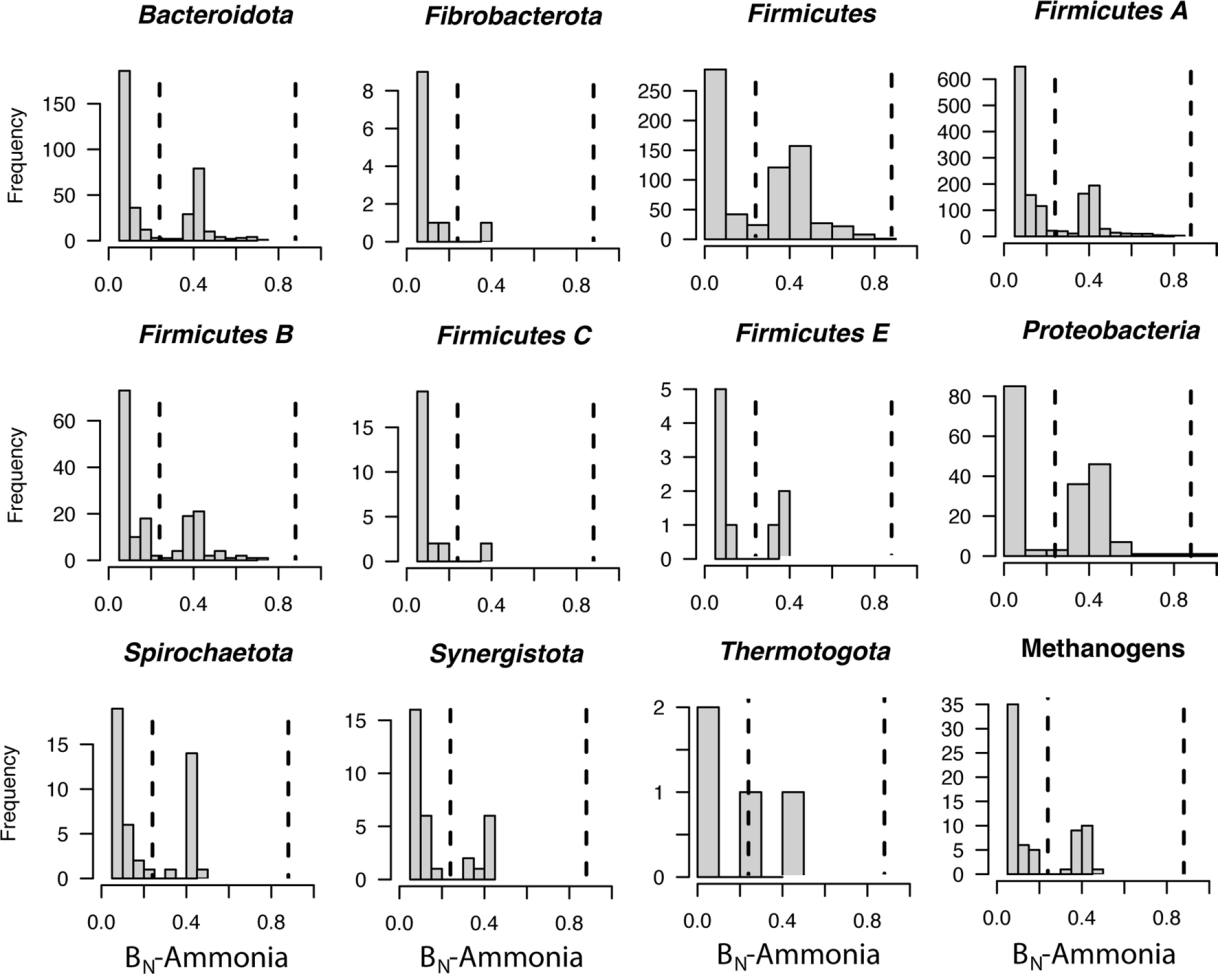
Histograms of the distribution of NH_3_ tolerant and sensitive taxa within 12 phyla that contribute important functions to anaerobic digestion. Dotted lines represent threshold values for sensitivity against, and preference for, NH_3_ based on null model testing. Taxa below B_N_-Ammonia 0.24 are sensitive, while taxa above 0.88 are enriched with NH_3_. Taxa above B_N_-Ammonia 0.24 were considered tolerant

The Hurlbert’s niche breadth in response to NH_3_ (B_N_-Ammonia) was calculated for each taxon to investigate the proportion of taxa that were sensitive (B_N_-Ammonia < 0.24) *vs*. tolerant (> 0.24). Figure 4 summarises these distributions for 12 phylogenetic groups important for AD. *Circa* 50% of *Firmicutes*, *Proteobacteria* and *Thermotogota* were tolerant to NH_3_. Only 30–40% of *Bacteroidota*, *Firmicutes* A, B, E, *Spirochaetota*, *Synergistota* and methanogens were tolerant to NH_3_. Members of the *Fibrobacterota* and *Firmicutes* C were particularly sensitive, with only *ca*. 10% of these taxa tolerant to NH_3_. Only very few taxa from these phyla were identified as preferring high NH_4_-N conditions (B_N_-Ammonia > 0.88), as two *Firmicutes* (*Limosilactobacillus* and *Erysipelothrix* sp.), and a proteobacterial *Pseudomonas* sp.

**Fig. 5:**
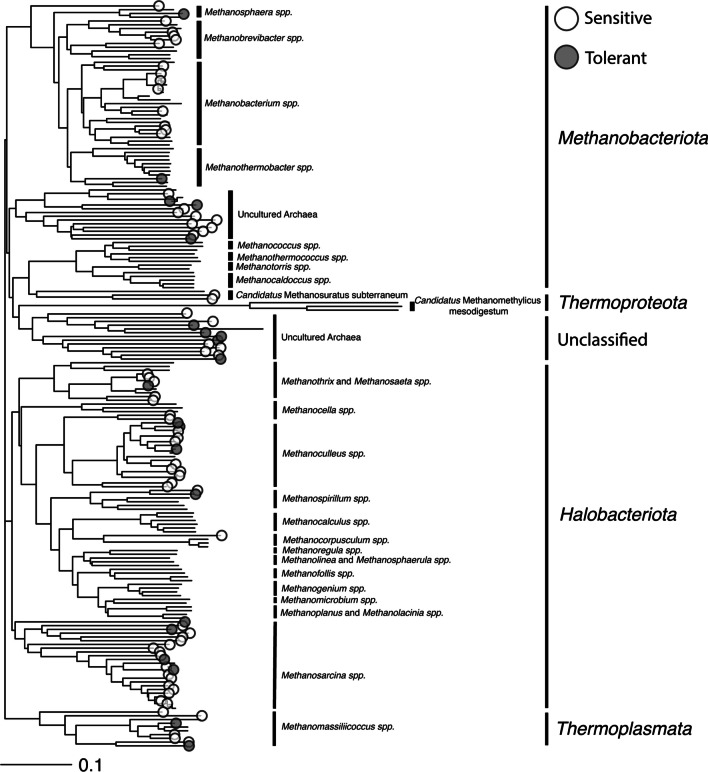
Neighbour-joining phylogenetic tree of methanogen ASVs detected in this study. Reference methanogens are leaves at the end of branches, while detected ASVs are highlighted as circles. These are coloured based on results of the B_N_-ammonia analyses, whereby white taxa were sensitive to NH_3_ and grey taxa were tolerant. Genera and phyla names of clusters are highlighted. The scale bar represents 10% variation in sequence identity

Hidden Markov Model–based analysis specific for methanogen ASVs was used to explore any potential phylogenetic trends for sensitivity vs. tolerance in this functional group. No specific genera were found to be tolerant, but rather select individuals from widespread methanogenic groups demonstrated tolerance (Fig. 5). Tolerant *Methanobacteriota* included a *Methanosphaera* and *Methanobacterium*, in addition to four unknown *Methanobacteriota*. Tolerant *Halobacteriota* included a *Methanothrix* (formerly *Methanosaeta*), three *Methanoculleus*, a *Methanospirillum*, two *Methanosarcina* and several unknown *Halobacteriota*. One tolerant ASV was distantly (*ca.* 90%) related to *Methanomassiliicoccus luminyensis* B10, and another as an unknown *Thermoplasmata*. Five tolerant ASVs were unclassifiable *Archaea*. As mentioned above, the majority of methanogens were sensitive to NH_3_, and no tolerant ASVs were identified from *Methanobrevibacter*, *Methanothermobacter*, *Methanococcus*, *Methanothermococcus*, *Methanocaldococcus*, *Methanocella*, *Methanocalculus*, *Methanocorpusculum*, *Methanoregula*, *Methanolinea*, *Methanosphaerula*, *Methanofollis*, *Methanogenium*, *Methanomicrobium*, *Methanoplanus*, *Methanolacinia* or *Candidatus* Methanosuratus, *Can*. Methanomethylicus or *Can*. Bathyarchaeota lineages.

### Relationships between tolerant taxa under varying NH_4_-N conditions

**Fig. 6:**
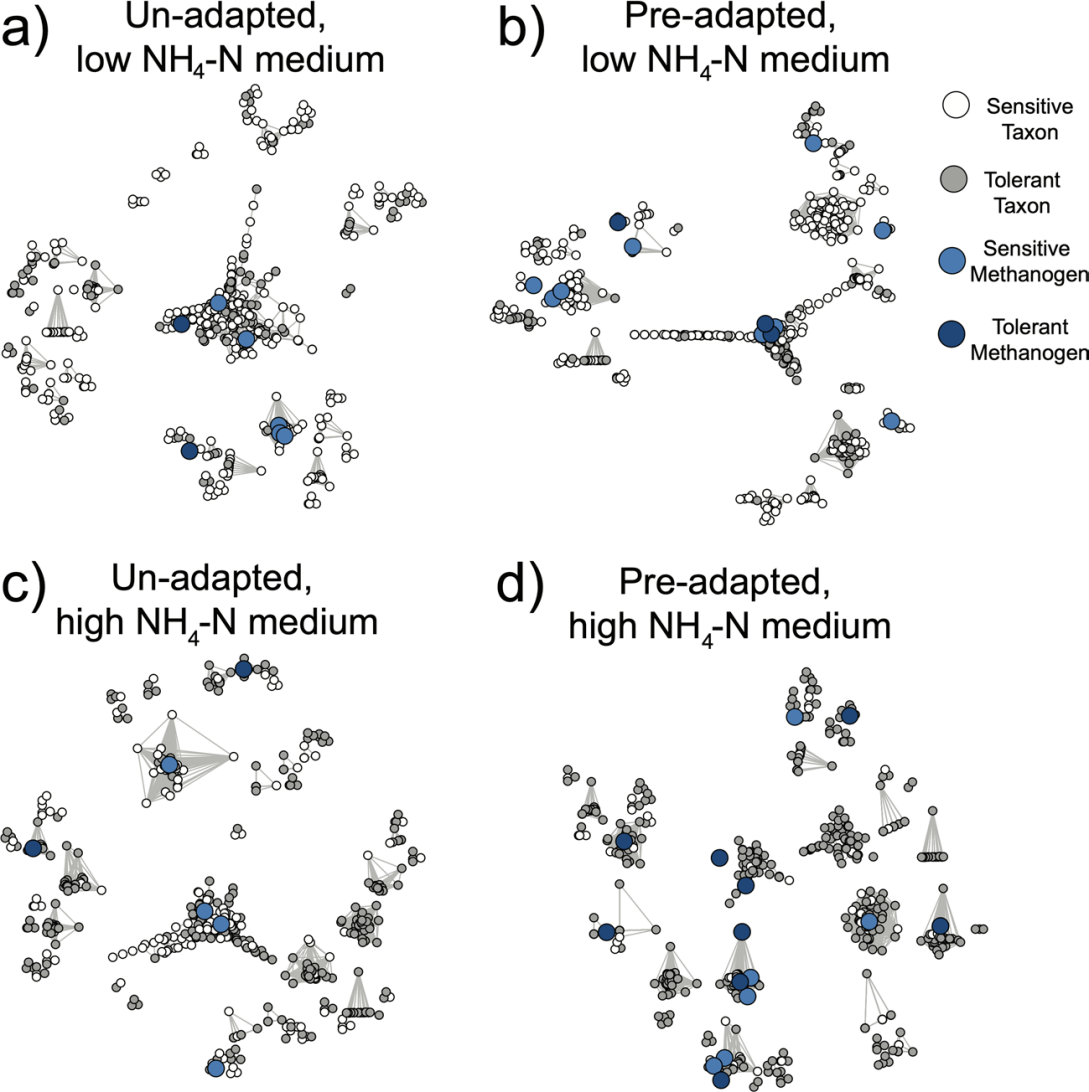
Network analyses of un- and pre-adapted communities under low NH_4_-N media (**a** and **b**, respectively), and un- and pre-adapted communities under high NH_4_-N media (**c** and **d**, respectively). Nodes represent the following: white = an NH_3_ sensitive taxon; grey = a tolerant taxon; light blue = a sensitive methanogen; dark blue = a tolerant methanogen. Edges represent significant positive Spearman correlations over time, indicating strong covarying trends in growth

Figure 6 compares covarying growth patterns between taxa under the four experimental combinations, over the 2-week incubation period. The majority of taxa from the un-adapted slurry, growing in low NH_4_-N medium, were sensitive to NH_3_ (75.9% of all taxa, white nodes). There were more sensitive methanogens than tolerant (75% of methanogens, light blue nodes) and these were associated with two separate network clusters. Once put under the pressure of the high NH_4_-N medium, more tolerant taxa appeared in the network (61.4% of all taxa, dark grey nodes) and individual clusters dominated by tolerant taxa emerged. However, only two of these clusters were associated with NH_3_ tolerant methanogens (dark blue nodes). The total number of methanogens was reasonably consistent, regardless of low or high NH_4_-N growth medium (seven vs. six, respectively).

When the pre-adapted community was grown under low NH_4_-N conditions, sensitive taxa dominated the network once again, with numbers reflecting that of the un-adapted community (74.3% of all taxa, white nodes). Sensitive methanogens (light blue nodes) formed associations with seven different network clusters and predominated over tolerant methanogens (80% of methanogens). Ammonia tolerant taxa from the pre-adapted community only persisted under high NH_4_-N growth medium, dominating the network (86.5% of all taxa, dark grey nodes). All individual clusters were dominated by NH_3_ tolerant taxa, with seven such clusters associated with their own tolerant methanogen (dark blue nodes). Sensitive methanogens persisted in this network (40% of methanogens, light blue nodes) and also formed tight associations with NH_3_ tolerant taxa. The total number of methanogens was once again consistent between networks, regardless of low or high NH_4_-N growth medium (both 15). Chi-square testing indicated that the differences between networks were driven by the total numbers of sensitive *vs*. tolerant taxa (*p* < 0.001). By comparison, the sensitivity and/or total numbers of methanogens did not vary between the networks (*p* = 0.12).

## Discussion

As the mechanism of NH_3_ toxicity is suspected to be non-specific, and AD is a community-scale process, this study sought to investigate the broad-scale impact of NH_3_ on biogas slurry prokaryotes and the importance of tolerance at the community-scale. It was suspected that a community sourced from a reactor running under ‘high’ NH_4_-N conditions would demonstrate some form of pre-adaptation to NH_3_, at least relative to one sourced from ‘low’ conditions, i.e. community adaptation to environmental legacy effects (Vass and Langenheder 2017). By incubating these two distinct communities under separate non-inhibitory (0.09 mg NH_3_ mL^−1^) and inhibitory (0.3 mg NH_3_ mL^−1^) growth conditions, it was possible to track each taxon’s response to NH_3_ over time and to evaluate their sensitivity. Specifically, it was hypothesised that (1) pre-adaptation would lead to improved methanogenesis under high NH_4_-N conditions; (2) that taxa involved in all functional steps of AD were affected by NH_3_, not just methanogens; and (3) that it is the continuation of co-operative ecological interactions between taxa, at the community-scale, that allows methanogenesis to continue. While the overall incubation time was relatively short (14 days), in practice, a sudden change to a high-protein substrate input can indeed result in rapid and prolonged increases in reactor NH_3_ concentrations within 1–2 weeks (Yirong et al. 2017). Even so, it should be emphasised that it is very unlikely that any adaptation by the prokaryote communities would occur under the conditions of this experiment. Rather, any such observable adaptations would have occurred in the biogas reactors from which they were sampled.

In line with the first hypothesis, the pre-adapted community performed methanogenesis under inhibitory concentrations of NH_3_ better than its un-adapted counterpart (Fig. 1). A commonly reported toxicity threshold is *ca*. 1.7 g TAN L^−1^ (Yenigün and Demirel 2013), whereby methanogenesis can shut down in un-adapted communities if there is a sudden, sharp increase in TAN above this. In this study, the un-adapted community came from a source below this threshold, while the pre-adapted community came from a reactor above it (Table 1). Many studies have demonstrated that it is possible to adapt mesophilic communities to TAN concentrations of 2 g TAN L^−1^ (Koster and Lettinga 1988), 3 g TAN L^−1^ (Hobson and Shaw 1976; Melbinger and Donnellon 1971), 4.5 g TAN L^−1^ (Yirong et al. 2017) and even up to 7 g TAN L^−1^ (Fotidis et al. 2013). The relatively short incubation time of 2 weeks was insufficient to see any potential for adaption (i.e. methanogenesis) in the un-adapted community. The presence of *ca*. 2 mL CH_4_ L^−1^ in the gas headspace at day 14 suggests that a longer incubation may have eventually resulted in improved methanogenesis. For the purposes of this study, the contrast in CH_4_ production between un-adapted and pre-adapted communities indicated that NH_3_ toxicity was successfully applied and that the following ecological responses could be associated with this.

When exposed to NH_3_-stress, the growth of over half of the un-adapted phyla was negatively impacted (Fig. 2). These impacted phyla are associated with numerous functionally important steps in the AD process, and their decreased abundance suggests dire consequences for CH_4_ production. For example, taxa within the *Acidobacteriota*, *Bacteroidota*, *Chloroflexota*, *Fibrobacterota*, *Firmicutes* A and *Planctomycetota* are essential for the anaerobic hydrolysis of cellulose, protein and lipids (Cai et al. 2016; Hug et al. 2013; Nobu et al. 2020) and any decrease in these functions required for the first step of AD will impact downstream process rates. Toward the other end of the AD process, the *Spirochaetota*, *Synergistota* and *Thermotogota* are essential secondary fermenters that oxidise fatty acids and/or form syntrophic interactions with hydrogenotrophic methanogens (i.e. formate or hydrogen transfer) (Nobu et al. 2020; Nobu et al. 2015). As has been well documented previously, methanogenic phyla can also be sensitive to NH_3_-toxicity (Lv et al. 2019; Moestedt et al. 2016; Ziganshin et al. 2016), and the *Halobacteriota*, *Methanobacteriota* and *Methanobacteriota* B from the un-adapted slurry were negatively impacted here.

In contrast, most of the phyla present in the pre-adapted high NH_4_-N slurry were unaffected by inhibitory concentrations of NH_3_ (Fig. 2). Some of the aforementioned phyla (e.g. *Acidobacteriota*, *Chloroflexota*, *Methanobacteriota* B, *Planctomycetota*, *Spirochaetota*) were either entirely absent from, or in very low abundance within, this community. Earlier pre-adaptation had likely already selected against the majority of NH_3_-sensitive individuals (Vass and Langenheder 2017), resulting in a poorly diverse (Table 1) yet NH_3_-robust community. Indeed, despite an inherently lower biodiversity, the high NH_4_-N community was still able to perform methanogenesis relatively well under non-inhibitory levels (Fig. 1). This suggests firstly that methanogenesis and biodiversity are decoupled in AD. Extensive functional redundancy within AD communities has been observed previously (Cai et al. 2016), and such ecological systems are typically robust to disturbance/stressors (Allison and Martiny 2008). It should be noted, however, that the communities in this study were provided a single substrate under batch culture conditions, and that biodiversity could be more valuable in long-term reactors fed varying, chemically-diverse substrates over time. Secondly, survival of NH_3_-tolerant taxa under inhibitory conditions came at the cost of a *ca*. 70% decrease in methanogenesis. This has also been observed previously within NH_3_ pre-adapted communities, and is thought to be due to the specific loss of aceticlastic methanogens and/or syntrophic fatty acid oxidisers (Fotidis et al. 2013; Lv et al. 2019; Wang et al. 2015). As these and other important functional groups from the pre-adapted community were not negatively affected, alternative explanations for the decreased efficiency in methanogenesis are warranted (discussed further below).

The taxonomic resolution of Fig. 2 is somewhat misleading as a single phylum may contain hundreds or even thousands of unique taxa. Therefore, this study also sought to investigate the distribution of NH_3_-tolerance amongst taxa with a particular focus on phyla that convey important functions for AD (Fig. 4). This was done through niche breadth analysis, whereby the response of each taxon’s abundance in relation to NH_3_ was calculated as a normalised ratio between 0 and 1 (Finn et al. 2020; Hurlbert 1978). While all phyla were predominantly comprised of sensitive taxa (B_N_-Ammonia < 0.24), they also contained some tolerant individuals (10–50%). This suggests that mechanisms for NH_3_ tolerance are widespread and non-specific. Such mechanisms could include removal of excess intracellular NH_3_ via increased synthesis and subsequent extracellular export of polyamines, e.g. putrescine and spermidine (Liu et al. 2021), increased glutamine/glutamate synthesis to remove intracellular NH_4_^+^ (Zimmer et al. 2000) or even simply increased export via transmembrane ammonia transporters, e.g. the ammonia transporter Amt (Li et al. 2007). A previous metagenomics study indicated that cell homeostasis under NH_3_ toxicity may be dependent on the combined presence of sodium/proton and potassium membrane transporters, and additional hydrogenase complexes, e.g. ferredoxin-dependent hydrogenase Ech (Yan et al. 2020). One of the very few taxa found to actually prefer high NH_3_ concentrations was a *Limosilactobacillus* sp., known to be strong producers of extracellular polysaccharides (Hossain 2022), and this may be yet another mechanism to protect the cell from diffusion of NH_4_^+^ via a thick hydrophobic layer. From a practical perspective, widespread, non-specific mechanisms of NH_3_ tolerance suggest the potential for adapting diverse, independent biogas reactors to high NH_4_-N. This seems plausible as many independent laboratories have successfully acclimated CH_4_ producing slurry communities in the past, even from diverse operating conditions (e.g. thermophilic and mesophilic) and substrate inputs (Fotidis et al. 2013; Hansen et al. 1998; Yirong et al. 2017).

Previous studies into NH_3_ inhibition have primarily focused on methanogenic *Archaea* (Borja et al. 1996; Fotidis et al. 2013; Lv et al. 2019; Moestedt et al. 2016; Ziganshin et al. 2016). Perhaps the only consistently enriched methanogen under NH_3_-stress is *Methanoculleus bourgensis* MS2, which has been shown to be both NH_3_-tolerant (Wang et al. 2015) and capable of improving CH_4_ yields when added to reactors as a bioaugmentation agent (Fotidis et al. 2014). In this study, several *Methanoculleus* spp. were found to be NH_3_-tolerant (100% identity to *M. bourgensis* MS2 and 98% identity to *M. palmolei* DSM4273, based on the V4 hypervariable region of the 16S rRNA gene). However, multiple other *Methanoculleus* spp. were found to be NH_3_-sensitive (e.g. *M. hydrogenitrophicus* HC, *M. thermophilus* CR-1, *M. olentangyi*). This inconsistency even at the genus level was reflected across the 92 putative methanogen ASVs identified, indicating that mechanisms for tolerance are not linked to methanogenic phylogeny. Rather, the kinds of physical mechanisms for tolerance described above may be continuously and independently emerging within taxa in response to NH_3_-stress, e.g. increased expression of polyamine synthesis under high NH_3_. Differential gene expression analysis of *M. bourgensis* MS2 as a model methanogenic taxon for NH_3_ tolerance could help to elucidate what specific adaptation(s) are required. In regard to the second hypothesis of this study, the broad-scale taxonomic and methanogen-specific analyses therefore suggested that methanogens were no more sensitive to NH_3_ than most other groups and that widespread, non-specific mechanisms for tolerance exist throughout slurry communities.

The final hypothesis sought to investigate an ecological explanation for the continuation of methanogenesis under inhibitory concentrations of NH_3_. Network analyses can give insight into the relationships between microbial taxa within communities, and/or their common response to a set of environmental conditions (Faust 2021). An interesting, yet perhaps unsurprising, common response was that NH_3_-tolerant taxa emerged to dominate communities under high NH_4_-N, regardless of the original slurry source (Fig. 6). This suggests that even the un-adapted community had the potential to become NH_3_-tolerant if the incubation period was extended, in line with the concept that potential mechanisms for tolerance are widespread and non-specific. Previous acclimation studies have reported that, even after 2–3 months without CH_4_ production, methanogenesis can recommence if the system pH is maintained between 7 and 8 (Koster 1986). Perhaps the only true inhibition occurs if acidosis (particularly acetic acid build-up) develops, whereby the entire microbial community dies (Lv et al. 2019). The key difference between the two communities under high NH_4_-N conditions was that the growth of tolerant methanogens was consistent with more tolerant taxa, visualised as connections between dark blue and grey nodes (Fig. 6). As explained above, methanogens perform the fourth step of AD, yet are dependent on other taxa to fulfil the other stages (Wirth et al. 2012). Here, the connections between tolerant methanogens and other tolerant taxa indicate the continuation of strong covarying growth, potentially as a result of maintained co-operative metabolic interactions (Faust and Raes 2016). In agreement with the third hypothesis, this could explain why at least some CH_4_ production continued here.

Finally worth noting was that, when the pre-adapted community was placed under low NH_4_-N conditions, the ratio of sensitive to tolerant taxa was actually reversed and the network was once more dominated by sensitive taxa. Such an outcome would be expected if there was a negative fitness associated with NH_3_-tolerance. For example, if increased polyamine synthesis is required for survival under high NH_3_, this may in fact be detrimental for growth under low conditions by simply being an unnecessary burden on general cell maintenance, or a loss of potential nitrogen for protein synthesis. An alternative explanation for the commonly observed decrease in methanogenesis under NH_3_-stress (of *ca*. 70% in this study) is therefore that tolerant taxa, regardless of their functional role in AD as hydrolysers, methanogens, etc., must invest carbon, nitrogen and energy into combating NH_3_-toxicity. This general reallocation of resources would come at the cost of decreased overall central carbon metabolism, which would ultimately lead to less CH_4_ production.

In conclusion, as the AD process requires co-operative metabolic interactions between many diverse taxa, this study sought to investigate effects of NH_3_ toxicity at the community-scale in a model laboratory system. By sourcing two distinct slurry communities, one pre-adapted and the other not, it was possible to compare and contrast responses to inhibitory concentrations of NH_3_. As expected, NH_4_-N pre-adaptation was necessary for methanogenesis under NH_3_-stress. While many phyla were shared between the distinct communities, those taxa from the pre-adapted slurry were more robust to NH_3_. Closer investigation into the distribution of NH_3_ tolerant vs. sensitive taxa found functional groups essential for all steps of AD, including methanogens, harboured tolerant individuals. By reconstructing ecological relationships via covarying growth patterns, continued methanogenesis under NH_3_-stress could be explained by enduring metabolic interactions between tolerant taxa, including methanogens, at the community-scale. While the specific physiological mechanisms that give rise to NH_3_ tolerance were not identified in this study, these results suggest that such mechanisms are widespread and unlinked to specific phylogeny. As NH_3_ inhibition during digestion of protein-rich substrates is a common problem, widespread tolerance of taxa has practical significance regarding the potential to adapt biogas reactors to NH_3_. Future studies should consider (meta)transcriptomic and (meta)proteomic approaches to confirm the nature of the actual mechanisms underlying NH_3_-tolerance.

### Supplementary information

ESM 1 (PDF 775 kb)

## Data Availability

Illumina MiSeq amplicon data has been uploaded to the European Nucleotide Archive as Bioproject: PRJEB61507.
