# Supplementary material for: Methanogenesis in biogas reactors under inhibitory ammonia concentration requires community-wide tolerance
Source: Appl Microbiol Biotechnol. 2023 Sep 6;107(21):6717–30. doi: 10.1007/s00253-023-12752-5 (PMC10567828; doi:10.1007/s00253-023-12752-5)
Supplement: Supplementary file 1 — (PDF 775 kb) [file 253_2023_12752_MOESM1_ESM.pdf]

Applied Microbiology and Biotechnology

Biogas methanogenesis under inhibitory ammonia depends on community-wide tolerance

Damien R. Finn[1], Lena Rohe[2], Sascha Krause[3], Jabrayil Guliyev[4], Achim Loewen[4], Christoph C. Tebbe[1]

[1]Thünen Institute for Biodiversity, Johann Heinrich von Thünen Institute, Braunschweig 38116, Germany;

[2]Thünen Institute for Climate-Smart Agriculture, Johann Heinrich von Thünen Institute, Braunschweig 38116, Germany;

[3]School of Ecological and Environmental Sciences, East China Normal University, 200062 Shanghai, China;

[4]Faculty of Resource Management, University of Applied Sciences and Arts (HAWK), 37085 Göttingen, Germany

Corresponding author: Damien R. Finn, Thünen Institute for Biodiversity, Johann Heinrich von Thünen Institute, Braunschweig 38116, Germany, damien.finn@thuenen.de.

Supplementary Material

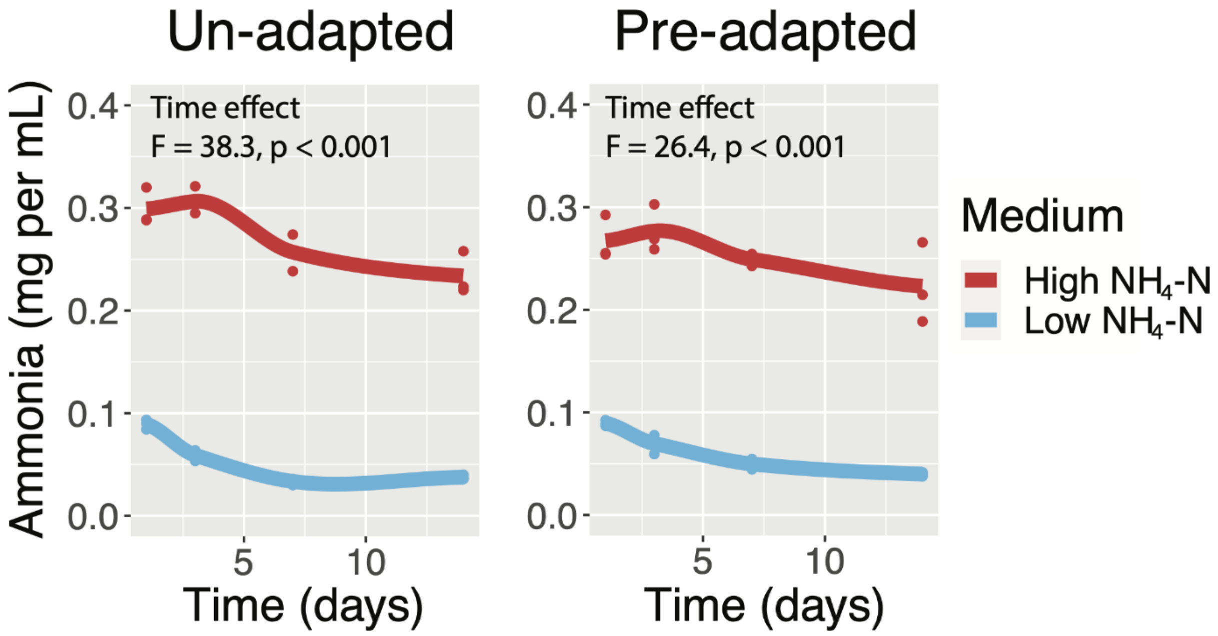

Supplementary Figure 1: Ammonia concentrations of low and high NH_4-N growth media over time. Trend lines represent best fit of local regression (loess). Analysis of variance results testing whether NH_3 changed over time are shown.

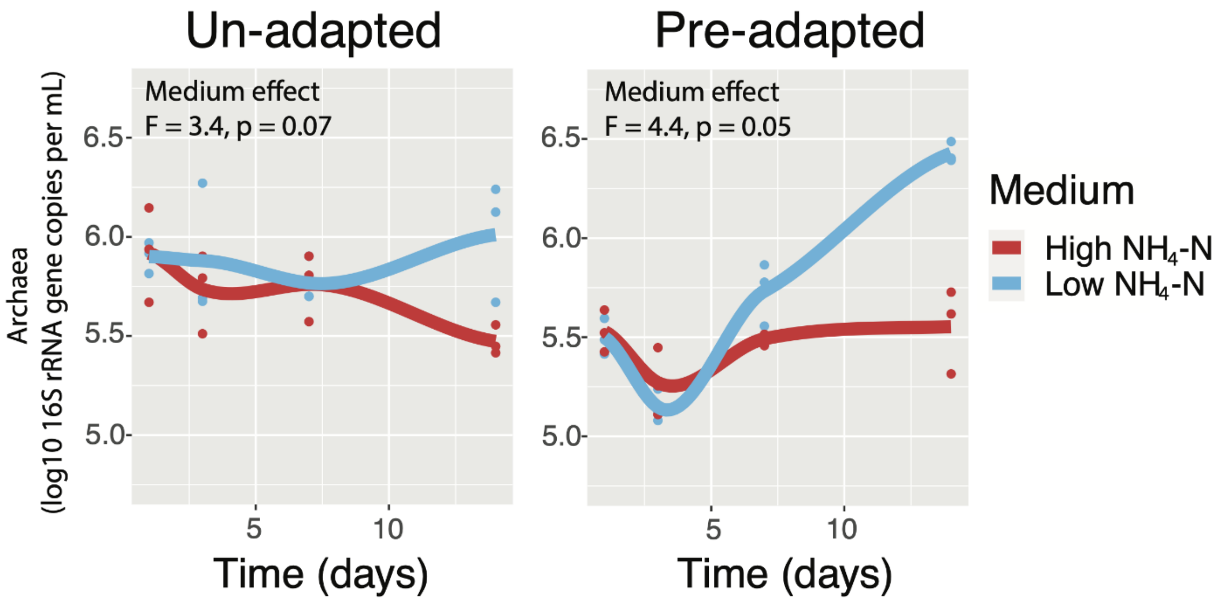

Supplementary Figure 2: Population sizes of Archaea in un- and pre-adapted communities over time. Trend lines represent best fit of local regression (loess). Analysis of variance results comparing an effect of low *vs.* high NH_4-N medium for each community are shown.

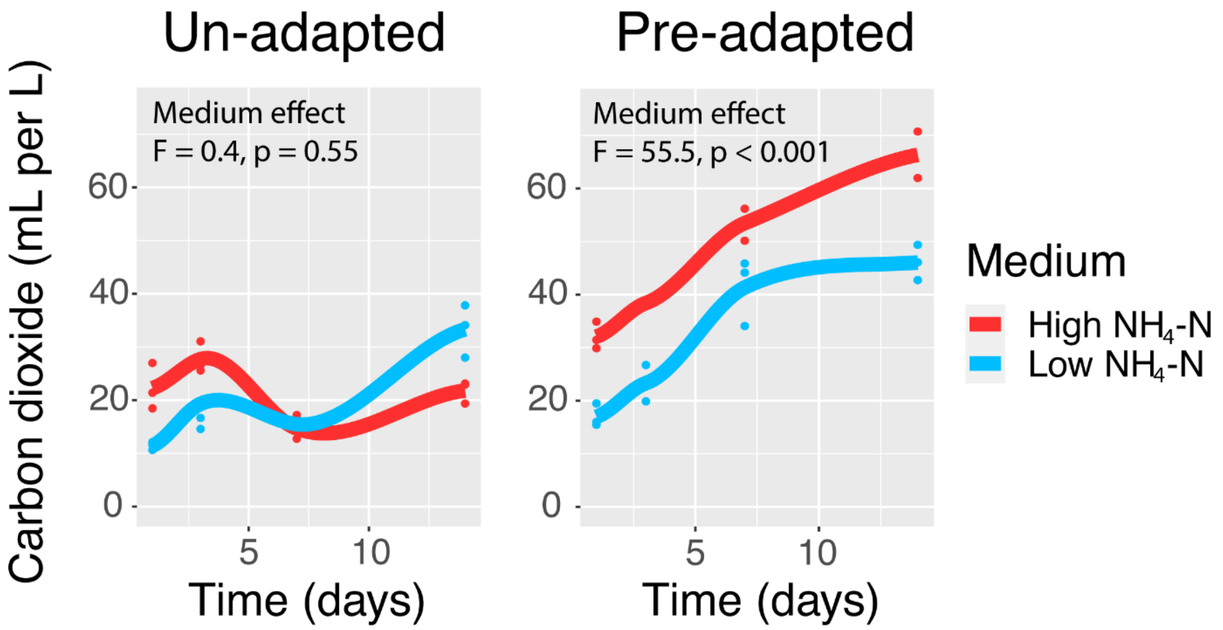

Supplementary Figure 3: Headspace carbon dioxide production by un- and pre-adapted communities over time. Trend lines represent best fit of local regression (loess). Analysis of variance results comparing an effect of low *vs*. high NH_4-N medium for each community are shown.

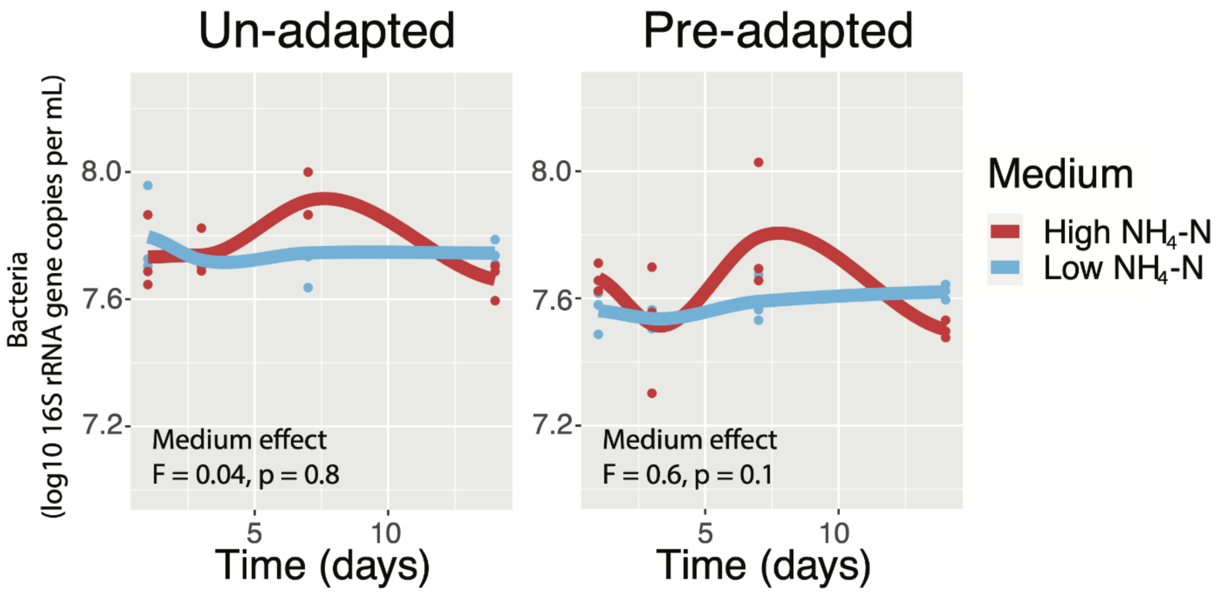

Supplementary Figure 4: Population sizes of Bacteria in un- and pre-adapted communities over time. Trend lines represent best fit of local regression (loess). Analysis of variance results comparing an effect of low *vs.* high NH_4-N medium for each community are shown.

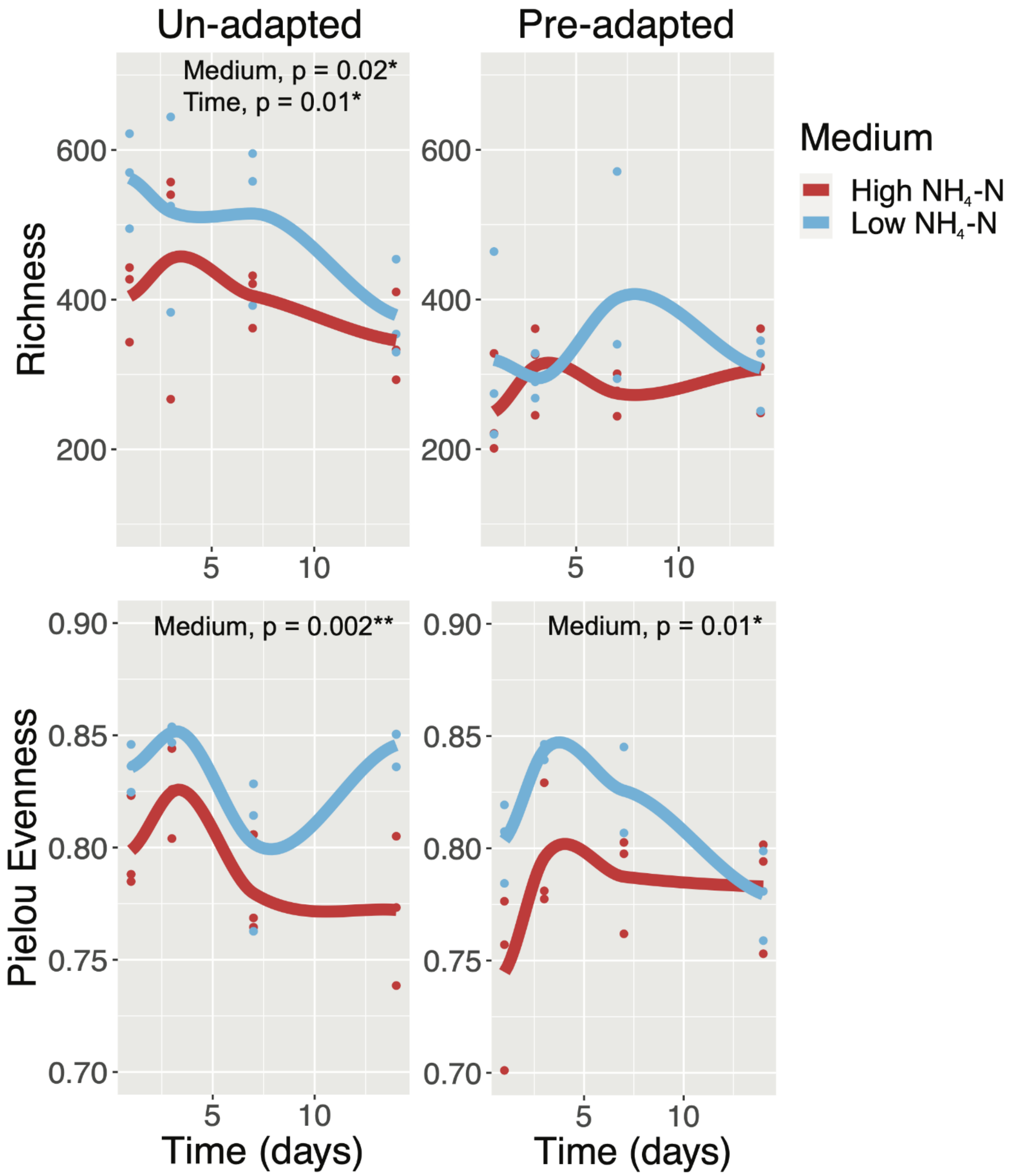

Supplementary Figure 5: Alpha-diversity as ASV Richness and Pielou Evenness of un- and pre-adapted communities over time. Trend lines represent best fit of local regression (loess). Analysis of variance results comparing either an effect of low *vs.* high NH_4-N medium or change over time for each community are shown.

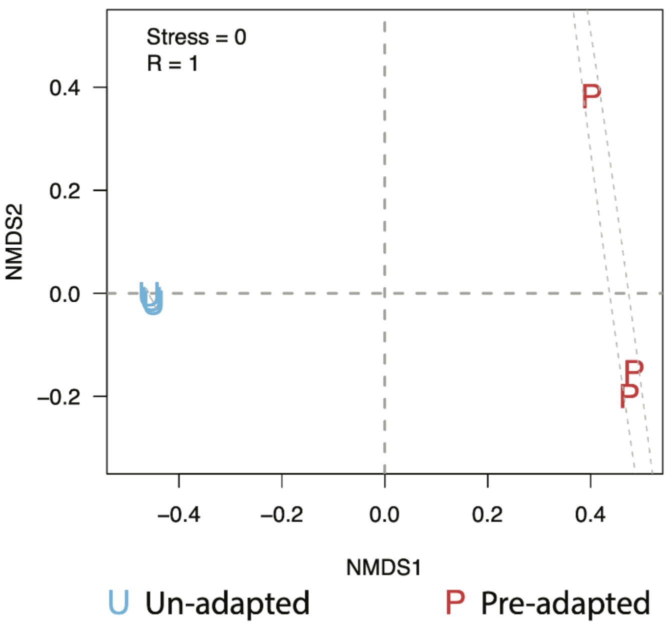

Supplementary Figure 6: Non-metric multidimensional scaling comparing the two source communities, one un- and one pre-adapted. Beta-diversity is based on Bray-Curtis transformation of absolute abundances of ASVs. Grey dotted lines represent 95% confidence intervals.

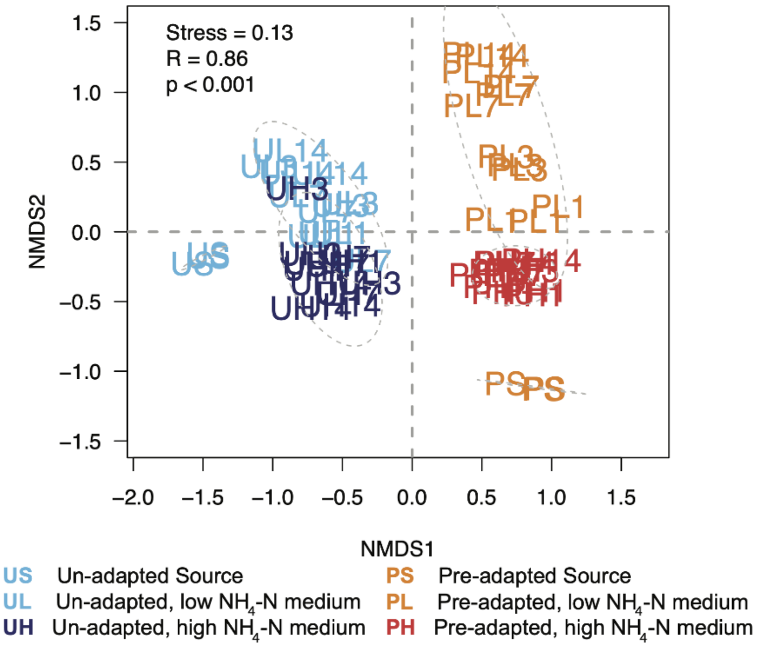

Supplementary Figure 7: Non-metric multidimensional scaling of (dis)similarity of all communities in this study. Beta-diversity is based on Bray-Curtis transformation of absolute abundances of ASVs. Numbers at the end of sample codes represent incubation day of sampling. Colours are based on growth medium and slurry source. Grey dotted lines represent 95% confidence intervals. Analysis of Similarity results testing for changes in community composition over time, between the growth media, are shown.
